# Integrated LC–MS and GC–MS-based untargeted metabolomics studies of the effect of azadirachtin on *Bactrocera dorsalis* larvae

**DOI:** 10.1038/s41598-020-58796-9

**Published:** 2020-02-10

**Authors:** You Zhou, De Qiang Qin, Pei Wen Zhang, Xiao Tian Chen, Ben Ju Liu, Dong Mei Cheng, Zhi Xiang Zhang

**Affiliations:** 1 Guangdong Biological Pesticide Engineering Technology Research Center, South China Agricultural University, Guangzhou, 510642 China; 2 Key Laboratory of Natural Pesticide and Chemical Biology of the Ministry of Education, South China Agricultural University, Guangzhou, 510642 China; 3 College of Agriculture and Biology, Zhongkai University of Agriculture and Engineering, Guangzhou, 510642 China

**Keywords:** Metabolomics, Entomology

## Abstract

Azadirachtin exhibits excellent bioactivities against several hundred arthropods. However, current knowlege of its biochemical effect on *B*. *dorsalis* larvae is not deep enough. In this study, integrated LC-MS and GC-MS-based untargeted metabolomics were used to analyze the changes of endogenous metabolites and the biochemical effects of azadirachtin on *B*. *dorsalis* larvae. Azadirachtin has excellent bioactivities against *B*. *dorsalis* larvae in this study, leading to a longer developmental duration, lower survival rate, and low pupa weight. The effect of azadirachtin was investigated on a total of 22 and 13 differentially abundant metabolites in the LC–MS and GC–MS-based metabolomics results, are selected respectively. Pathway analysis indicated that 14 differentially enriched metabolic pathways, including seven influential pathways, are worthy of attention. Further integrated key metabolic pathway analysis showed that histidine metabolism, d-glutamine and d-glutamate metabolism, biotin metabolism, ascorbate and aldarate metabolism, pentose and glucuronate interconversions, and alanine, aspartate and glutamate metabolism in *B*. *dorsalis* larvae are significantly relevant pathways affected by azadirachtin. Although extrapolating the bioactivity results in this study to the practical project of *B*. *dorsalis* pest management in the field has limitations, it was found that azadirachtin has a significant effect on the primary metabolism of *B*. *dorsalis* larvae.

## Introduction

*Bactrocera dorsalis* is a destructive polyphagous and invasive insect pest of tropical and subtropical fruits and vegetables; this oriental fruit fly has been found to attack many types of commercial fruits and a wide variety of agricultural products^1^. Azadirachtin exhibits excellent bioactivities against agricultural, forestry, medical, and veterinary arthropods^2–4^. However, studies on the effects of azadirachtin on *B*. *dorsalis* are scarce. Azadirachtin is the main active ingredient in neem. It was reported that neem derivatives are ineffective when used as toxic bait for tephritid fruit flies^5^. Several studies reported that neem seed kernel extracts and azadirachtin deters oviposition of *B*. *dorsalis* adults^6,7^. Neem leaf dust significantly reduced the longevity and fertility of *B*. *dorsalis* adults by blocking ovarian development^8^. Neem extract could effectively reduce the fecundity, fertility, and post-embryonic development of freshly emerged *B*. *dorsalis* flies^9^. However, we found no previous studies in the literature on the activity of azadirachtin against the larvae of *B*. *dorsalis*.

The biological effects of azadirachtin include impacts on egg-laying behavior, feeding behavior, growth and metamorphosis, reproduction, activity, and histopathology^10^. The mode of action of azadirachtin against lepidopteran insects can be explained, in part, by effects on digestive enzymes, NADPH cytochrome reductase, and cholinesterase^11^. The physiological effects of azadirachtin include direct inhibition of cell division and protein synthesis^12^. The indirect effects of blocking the release of morphogenetic peptide hormones (PTTH and allatostatins) causes disruption of molting hormone from the prothoracic glands and juvenile hormone from the corpora allata^12^. Transcriptomics analysis to investigate growth inhibition in *Drosophila* larvae after exposure to azadirachtin showed that 28 genes are significantly up or down regulated, with genes involved in starch and sucrose metabolism, defense response, signal transduction, instar larval or pupal development, and chemosensory behavioral processes were affected^13^. The 2DE proteomics analysis of azadirachtin showed that 21 proteins were differentially expressed, which involved cytoskeletal organization, transcription and translation, hormonal regulation, and energy metabolism^14^.

Azadirachtin can also have effects at the biochemical level by impacting insect endogenous metabolites. Azadirachtin could interfere with serotonin pools in the neuroendocrine system of locusts^15^. It significantly decreased the lipids levels in the fat body, hemolymph, and ovary of *Atractomorpha crenulata* and the amino acid content in the fat body, testes, seminal vesicle, and MARGs of *Odontopus varicornis*^16,17^. Azadirachtin was also found to severely reduce protein, glycogen, and lipid contents of *Plodia interpunctella*^18^. The levels of cholesterol, uric acid, urea, and glucose decreased in azadirachtin-treated larvae of *Hyphantria cunea* compared with the control^19^. Quantities of fatty acids and their relative composition in Asian corn borer larvae were significantly affected by azadirachtin at 0.1–10 ppm^20^. The protein, lipid, and glucose contents decreased, whereas uric acid increased when *Glyphodes pyloalis* larvae were fed with neem-treated mulberry leaves^21^. These studies clearly showed that azadirachtin can affect various biochemical compounds, such as carbohydrates, fatty acids, amino acids, cholesterol, uric acid, and urea. Such studies focused mainly on a few biochemical metabolites, and no further analysis was performed to determine the biological importance of these molecular changes.

Metabolomics, an important part of systems biology, identifies the entire profile of detectable metabolites contained in a biological system; it has also been used to reveal alterations in the endogenous metabolite levels that may result from disease processes, drug toxicity, or gene function^22^. In addition, metabolomics is a powerful bioanalytical tool and has been widely used in insect science, such as in the discovery of pesticide modes-of-action^23^, the pupal diapause of the cotton bollworm^24^, radiation-induced insect sterility technique^25^, and research on the Asian citrus psyllid *Diaphorina citri*^26^.

Hence we tested the bioactivities of azadirachtin against *B*. *dorsalis* larvae in this study. Thereafter, we introduced an approach of integrated untargeted metabolomics using UPLC–QTOF-MS and GC–Q-MS to explore the changes in endogenous metabolites and the potential biological implications.

## Results

### Azadirachtin bioactivities against *B*. *dorsalis* larvae

**Figure 1:**
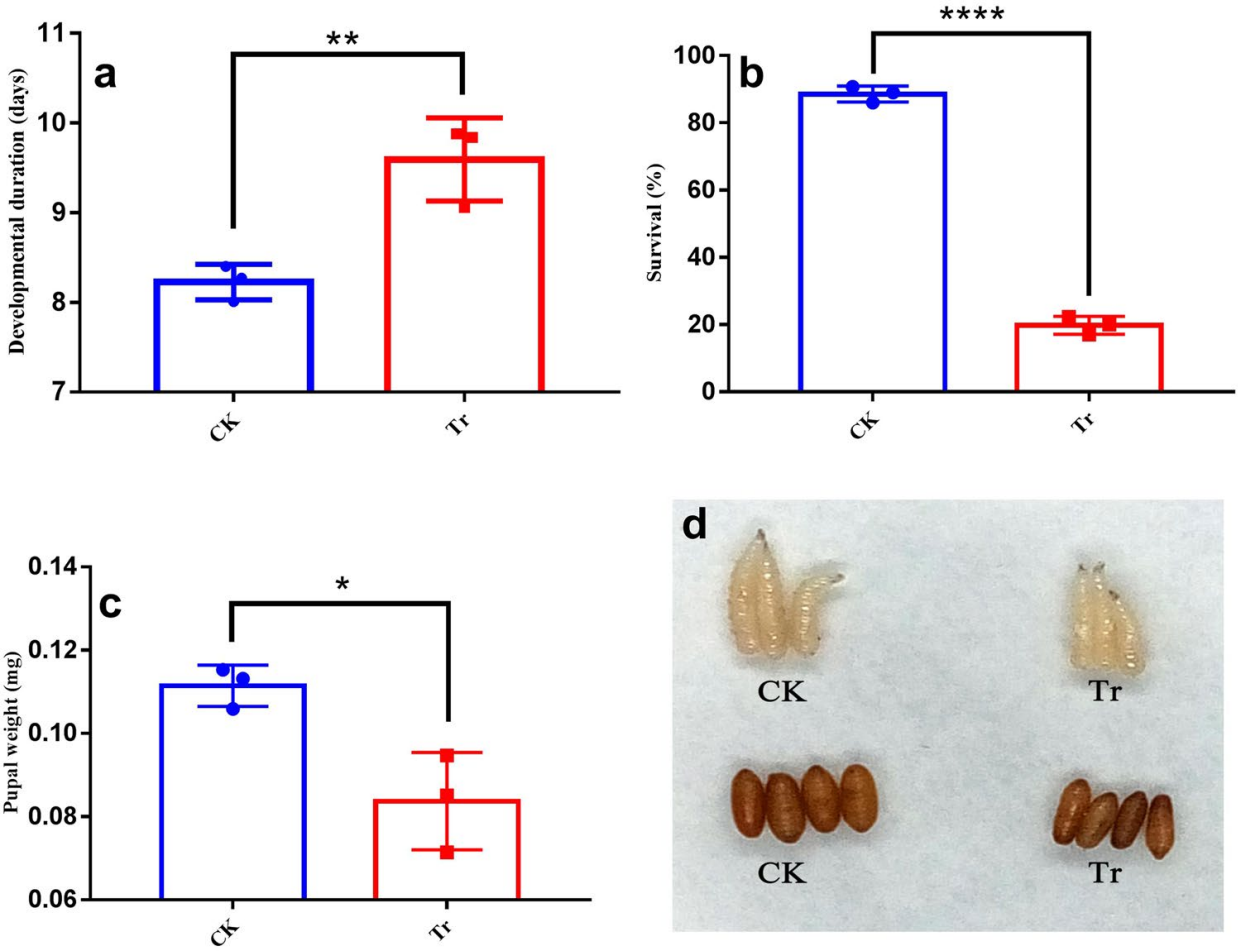
Bioactivities of azadirachtin against *B*. *dorsalis* larvae. Data were expressed as the mean ± SE. * Indicates *P* < 0.05, ** indicates *P* < 0.01, and **** indicates *P* < 0.001.

Azadirachtin was found to exhibit significant bioactivities towards *B*. *dorsalis* larvae (Fig. 1d). As shown in Fig. 1a, with regard to the developmental duration, 9.59 ± 0.27 days in the treatment (Tr) group was significantly longer than 8.23 ± 0.11 days in the control (CK) group (*P* < 0.01). As shown in Fig. 1b, in terms of survival, 19.78 ± 1.5% in the Tr group was significantly lower than 88.56 ± 1.4% in the CK group (*P* < 0.001). As shown in Fig. 1c, pupal weight, 0.084 ± 0.007 mg in the Tr group was significantly lower than 0.112 ± 0.003 mg in the CK group (*P* < 0.05).

### Metabolic profiles analyzed by GC–MS and LC–MS

**Figure 2:**
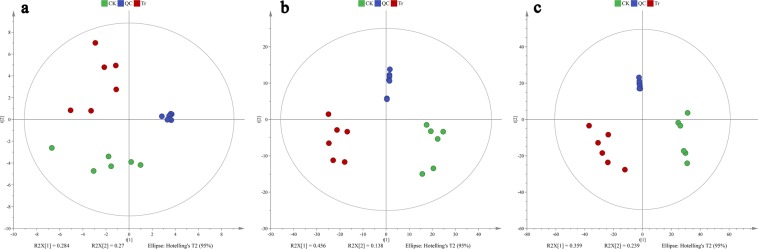
PCA score plots derived from (**a**) GC–MS, (**b**) negative ion mode (ESI−) and (**c**) positive ion mode (ESI+) in LC–MS metabolite profiles of *B*. *dorsalis* larvae.

The unsupervised principal component analysis (PCA) was used to check the quality of the data from the GC–MS and LC–MS analyses. They showed that all CK, Tr, and QC (quality control) samples were within the 95% Hotelling’s T-squared ellipse and significantly separated into clusters. That is no outlier was found among these samples. In the GC–MS analysis, the first principle component (PC1) and second principle component (PC2) explained 28.4% and 27% of the total variance of all samples (Fig. 2a). In ESI− mode, the PC1 and PC2 explained 45.6% and 13.8% of the total variance of all samples (Fig. 2b). In ESI+ mode, the PC1 and PC2 explained 35.9% and 23.9% of the total variance (Fig. 2c).

**Figure 3:**
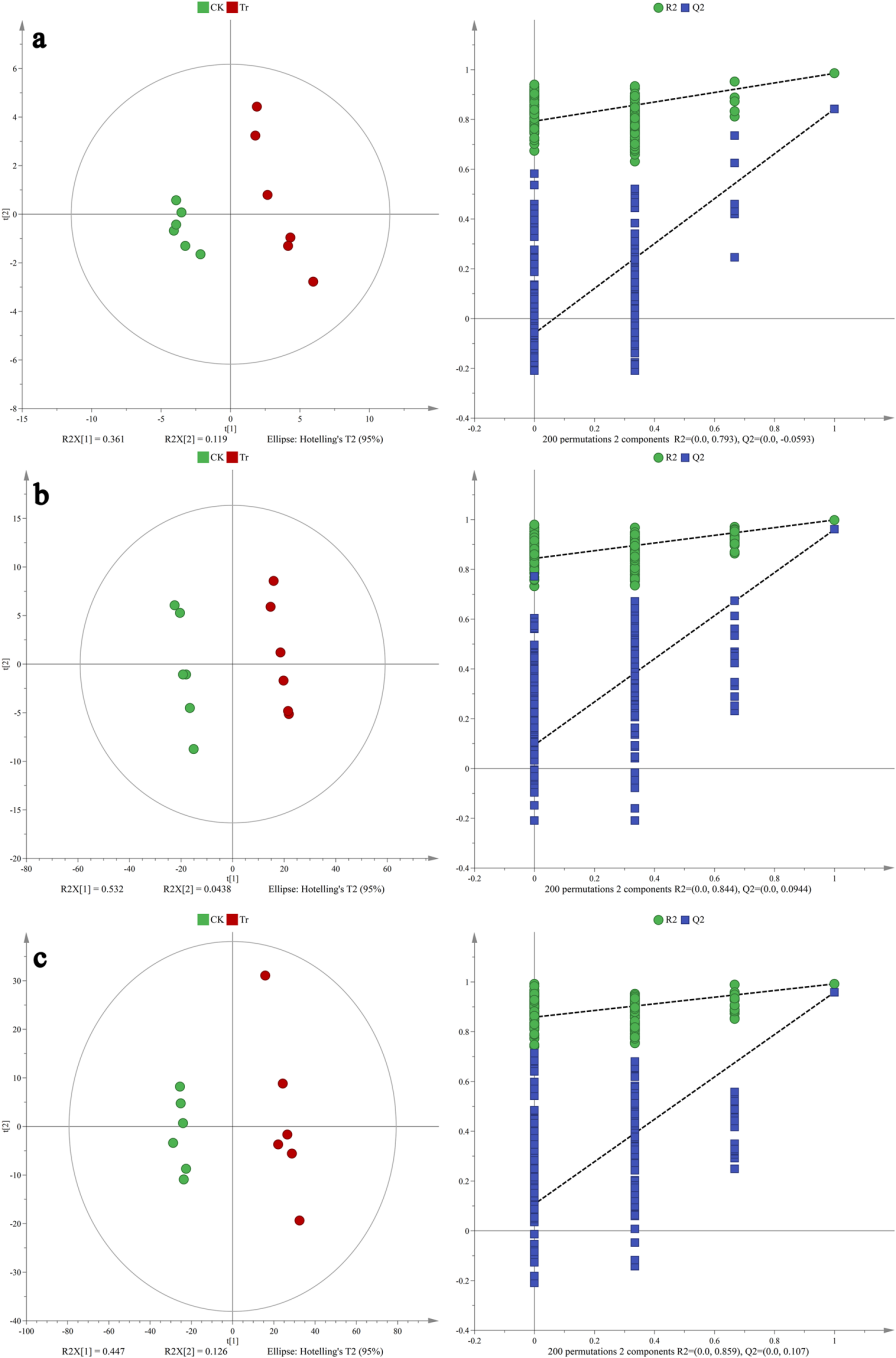
PLS-DA score plots (left) with corresponding permutation test plots (right) derived from (**a**) GC–MS, (**b**) negative ion mode (ESI−) and (**c**) positive ion mode (ESI+) in LC–MS metabolite profiles of *B*. *dorsalis* larvae.

The supervised partial least squares discrimination analysis (PLS-DA) was performed to identify the metabolites responsible for the separation between control and azadirachtin-exposed groups. The CK and Tr groups in these PLS-DA models were inside the 95% Hotelling’s T-squared ellipse and showed clear separation (Fig. 3). The 7-folds internal cross validation and 200 times permutation test were further conducted to assess these models’ predictive accuracy and statistical significance. In the GC–MS analysis, the parameters of PLS-DA model’s predictive accuracy were R^2^X_cum_ = 0.48, R^2^Y_cum_ = 0.985, and Q^2^Y_cum_ = 0.843; with its corresponding statistical significance were R^2^ = 0.793 and Q^2^ = −0.0593 (Fig. 3a). In ESI− mode, the parameters of PLS-DA model’s predictive accuracy were R^2^X_cum_ = 0.576, R^2^Y_cum_ = 0.999, and Q^2^Y_cum_ = 0.96; with its corresponding statistical significance were 0.844 and 0.0944 (Fig. 3b). In ESI+ mode, the parameters of PLS-DA model’s predictive accuracy were R^2^X_cum_ = 0.573, R^2^Y_cum_ = 0.992, and Q^2^Y_cum_ = 0.959; with its corresponding statistical significance were 0.859 and 0.107 (Fig. 3c). According to the criteria that if all blue Q^2^ values to the left are lower than the original points to the right or if the blue regression line of the Q^2^ points intersects the vertical axis at or below zero^27^, these PLS-DA models exhibited a low risk of overfitting. The above results indicated that these PLS-DA models could identify the differentially enriched metabolites between CK and Tr groups.

### Changed metabolites in *B*. *dorsalis* larvae between the CK and the Tr groups

**Figure 4:**
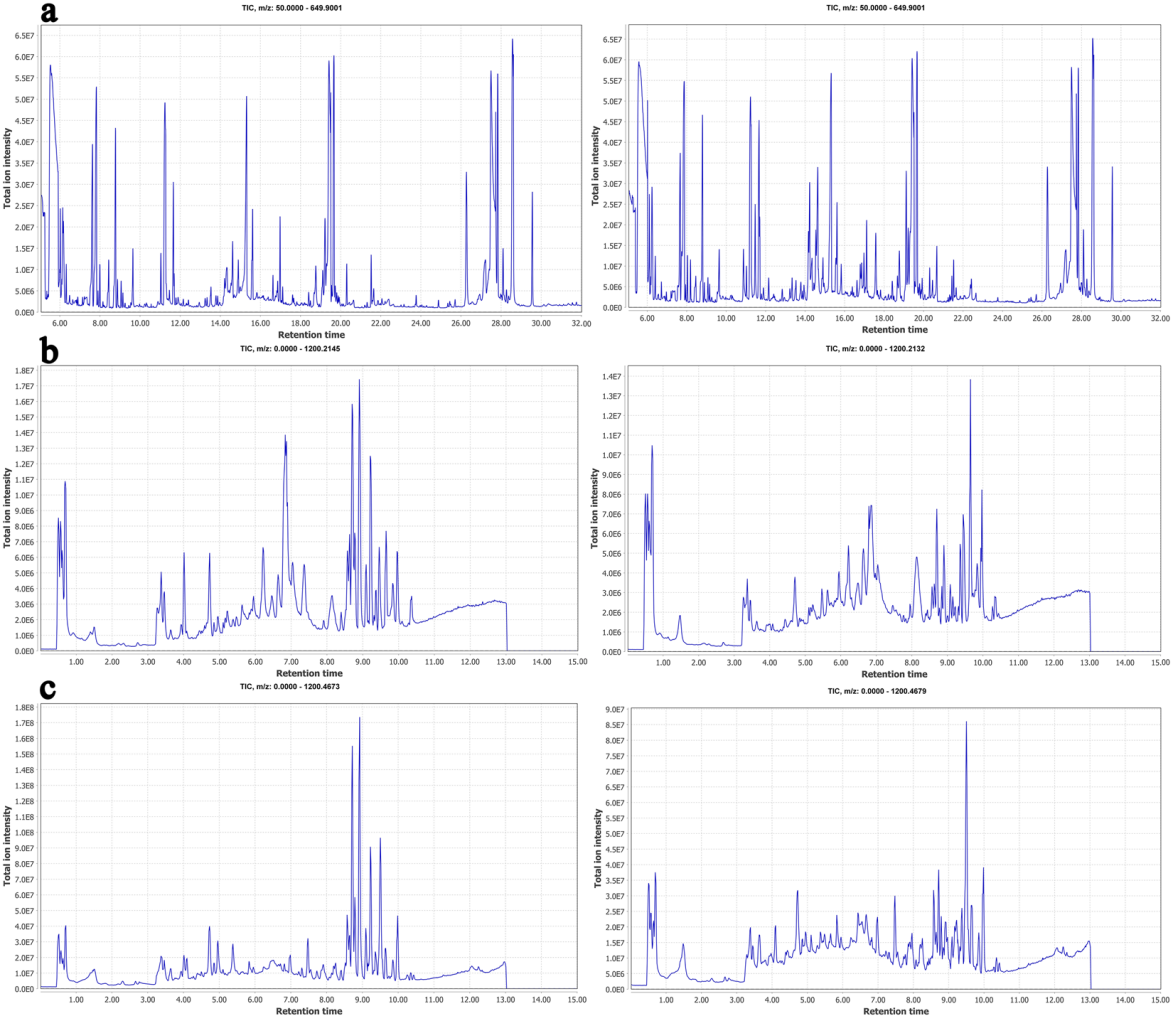
Typical TIC of *B*. *dorsalis* larvae tissue extracts obtained from (**a**) GC–MS, (**b**) negative ion mode (ESI−) and (**c**) positive ion mode (ESI+) in LC–MS. Left plots were CK samples, and right plots were Tr samples.

The representative GC–MS and LC–MS total ion chromatograms (TICs) of *B*. *dorsalis* larvae tissue samples are shown in Fig. 4. The shape and quantity of peaks between the CK and the Tr groups varied. Approximately 7328 and 13,375 metabolite peaks were deconvoluted in ESI− and ESI+ mode of LC–MS. By contrast, 415 metabolite peaks were deconvoluted in GC–MS. These deconvoluted data were further processed through missing value imputations, filtering, and normalization in MetaboAnalyst 4.0^28^. A total of 1979 and 3904 remaining peaks in ESI− and ESI+ modes in LC–MS and 235 remaining peaks in GC–MS were further annotated using references in existing databases. After the by-products in GC–MS and the exogenous compounds in LC–MS were removed, the differentially abundant metabolites were selected according to the VIP values from the PLS-DA model (VIP > 1.2) and the corrected *P* values from Student’s *t*-test (*q* value < 0.05). Table 1 shows that 15 of the 22 differentially abundant metabolites were upregulated in the LC–MS analysis. Table 2 illustrates that two of the 13 differentially abundant metabolites were downregulated in the GC–MS analysis. As shown in Tables 1 and 2, ten amino acids and derivatives of the differentially abundant metabolites were the most differentially abundant metabolites, followed by seven carbohydrates, six lipids, six nucleosides, three organic acids, and two vitamins and cofactors.

**Table 1:** Identification of differentially abundant metabolites in ESI+ and ESI− mode in LC–MS between the CK and the Tr groups.

Mode	Classification	RT (min)	Precursor MZ	Precursor Type	VIP	*q* value	Fold change	Regulation	Theoretical mass	Formula	Name
ESI+	Amino acids and derivatives	1.89	190.9997	[M + H]+	1.38	9.76E-04	1.171	Up	190.1106	C_11_H_14_N_2_O	5-Methoxytryptamine; 5-MeOT
		3.46	118.0647	[M + H]+	1.48	1.46E-04	0.852	Down	117.0790	C_5_H_11_NO_2_	Norvaline; D-Norvaline
		3.48	209.0921	[M + H]+	1.46	3.46E-04	0.865	Down	208.0848	C_10_H_12_N_2_O_3_	Kynurenine; L-Kynurenine
		3.73	178.0494	[M + H]+	1.23	6.73E-04	1.107	Up	177.0460	C_6_H_11_NO_3_S	N-Formylmethionine
		3.78	105.0699	[M + H]+	1.22	1.58E-03	1.119	Up	104.0586	C_3_H_8_N_2_O_2_	L-2,3-Diaminopropionic acid
		3.94	238.0355	[M + H]+	1.21	6.67E-03	0.879	Down	237.0307	C_7_H_11_NO_6_S	S-succinylcysteine
		7.78	156.0763	[M + H]+	1.26	1.03E-04	1.121	Up	155.0695	C_6_H_9_N_3_O_2_	Histidine; L-Histidine
		8.91	148.0602	[M + H]+	1.62	2.40E-03	1.331	Up	147.0532	C_5_H_9_NO_4_	Glutamic acid; L-Glutamic acid
		9.85	396.3479	[M + H]+	1.29	8.76E-04	1.152	Up	395.3399	C_24_H_45_NO_3_	N-Oleyl-Leucine
		9.89	430.3321	[M + H]+	1.34	2.04E-04	1.157	Up	429.3243	C_27_H_43_NO_3_	N-Oleoyl-Phenylalanine
	Nucleosides, nucleotides and derivatives	0.57	493.0011	[M + H]+	1.32	1.37E-04	1.170	Up	491.9848	C_10_H_15_N_4_O_13_P_3_	2′-Deoxyinosine-5′-triphosphate trisodium salt; dITP
		0.57	508.9798	[M + H]+	1.27	3.47E-05	1.153	Up	507.9798	C_10_H_15_N_4_O_14_P_3_	Inosine-5′-triphosphate trisodium salt; ITP
		1.30	169.0356	[M + H]+	2.14	3.69E-04	0.752	Down	168.0283	C_5_H_4_N_4_O_3_	Uric acid; Urate
		3.36	252.1096	[M + H]+	1.32	8.64E-04	1.184	Up	251.1018	C_10_H_13_N_5_O_3_	2′-Deoxyadenosine; Deoxyadenosine
		4.50	590.0901	[M + H]+	1.57	9.39E-03	0.760	Down	589.0822	C_16_H_25_N_5_O_15_P_2_	Adenosine 5′-diphospho-glucose; ADP-glucose
	Lipids and lipid-like molecules	5.20	204.1238	[M + H]+	1.54	1.46E-03	1.249	Up	203.1158	C_9_H_17_NO_4_	Acetyl-L-Carnitine; O-Acetylcarnitine
		6.66	862.6348	[M + H]+	1.33	5.94E-04	1.149	Up	861.6177	C_46_H_87_NO_13_	C16 Lactosyl Ceramide (d18:1/16:0)
		7.58	383.3262	[M + H]+	1.39	1.93E-04	1.170	Up	382.3236	C_27_H_42_O	Cholest-4,6-Dien-3-One
		9.22	510.3535	[M + H]+	1.50	1.03E-03	0.825	Down	509.3481	C_25_H_52_NO_7_P	1-heptadecanoyl-2-hydroxy-sn-glycero-3-phosphocholine
	Vitamins and cofactors	1.30	262.1279	[M + H-H2O]+	1.51	5.87E-05	1.176	Up	244.0882	C_10_H_16_N_2_O_3_S	Biotin; Vitamin H
ESI−	Nucleosides, nucleotides and derivatives	0.73	167.0202	[M − H]−	1.96	3.43E-04	0.725	Down	168.028336	C_5_H_4_N_4_O_3_	Uric acid; Urate
	Carbohydrates and carbohydrate conjugates	3.79	326.1265	[M − H]−	1.66	6.50E-05	1.366	Up	327.0954	C_14_H_17_NO_8_	Acetaminophen glucuronide

**Table 2:** Identification of differentially abundant metabolites in GC–MS between the CK and the Tr groups.

Classification	Metabolites	VIP	*q* value	Fold change	Regulation	Retention time	Retention index	RI-RI (lib)	Match factor
Carbohydrates and carbohydrate conjugates	Erythritol	2.35	3.63E-09	0.828	Down	14.44	1509.7	—	87
	Threitol; D-Threitol	2.35	1.82E-09	0.828	Down	14.54	1517.6	2.6	91
	Xylulose; D-Xylulose	1.26	3.62E-04	1.060	Up	16.63	1694.5	34.5	88
	Arabitol; D-Arabitol	2.35	1.81E-09	0.816	Down	17.11	1735.0	—	96
	Xylitol	2.35	1.36E-09	0.816	Down	17.17	1739.7	−13.9	87
	Galactitol	1.25	5.13E-04	0.943	Down	19.93	1971.6	−10.2	91
Organic acids and derivatives	Succinic acid; Succinate	1.55	4.80E-03	0.870	Down	11.70	1313.2	9.5	92
	2,3-Dihydroxy-2-methylpropanoic acid	1.41	4.61E-05	0.936	Down	11.90	1326.1	—	88
	Malic acid; Malate	2.63	3.46E-03	0.746	Down	14.21	1493.0	−45.2	92
Lipids and lipid-like molecules	Octadecadienoic acid methyl ester, 9,12-(Z,Z)-, n-	2.69	2.63E-08	1.285	Up	21.18	2082.2	−7	84
	Octadecadienoic acid, 9,12-(Z,Z)-	1.24	1.54E-02	0.936	Down	22.37	2196.8	—	83
Nucleosides, nucleotides and derivatives	UDP-N-acetylglucosamine	1.42	2.67E-04	0.930	Down	18.41	1840.6	22.6	88
Vitamins and cofactors	Myo-Inositol	1.65	2.66E-05	0.920	Down	20.06	1984.4	37.2	92

### Metabolic pathway of differentially abundant metabolites

**Figure 5:**
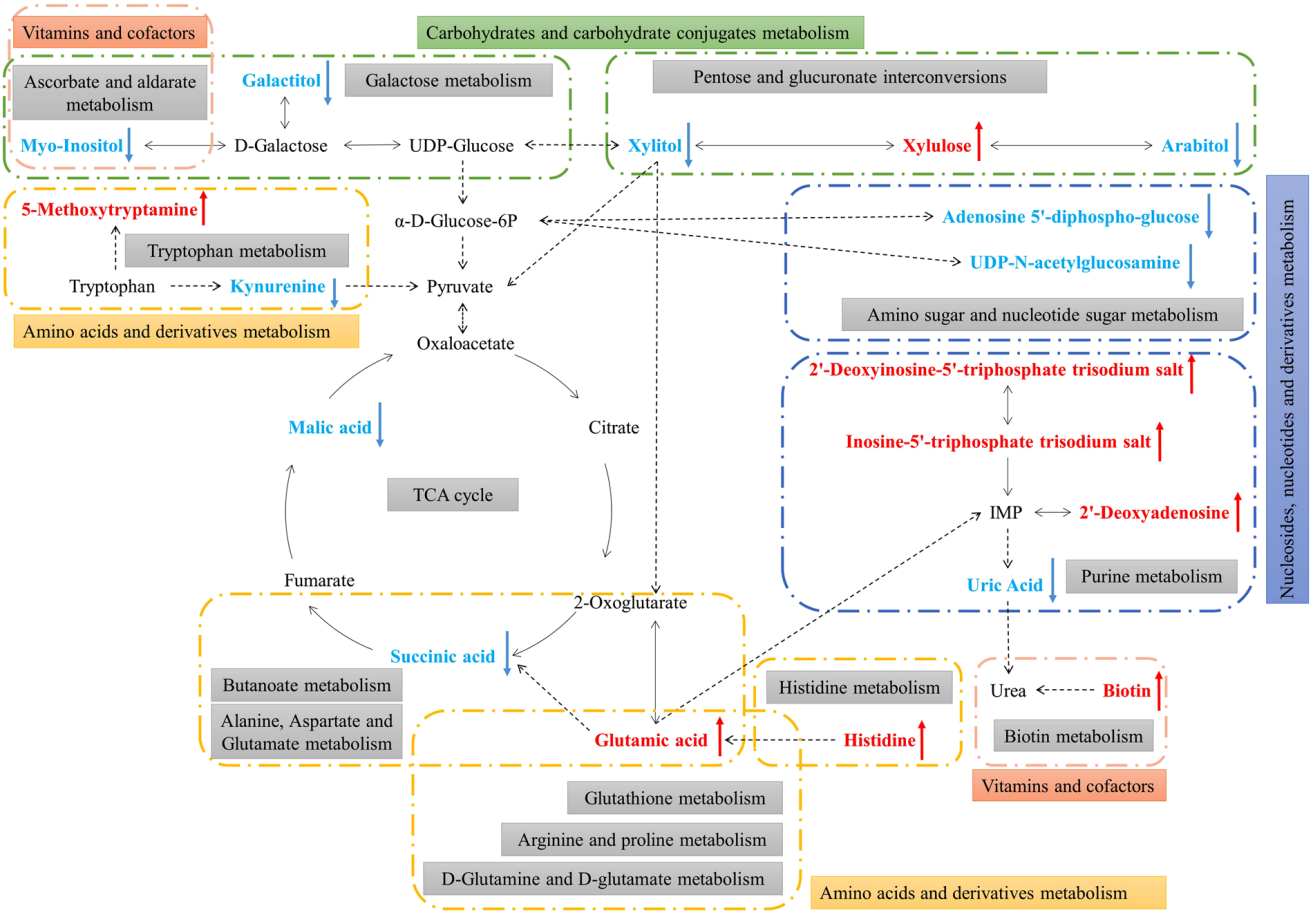
Schematic overview of the primarily affected metabolic pathways in *B*. *dorsalis* larvae due to feeding with the azadirachtin diet. The red characters indicate increased metabolites, and the green ones indicate decreased metabolites.

The KEGG pathway analysis of differentially abundant metabolites was performed by MetaboAnalyst 4.0 to identify the disturbed metabolic pathways caused by feeding with the azadirachtin diet. A schematic overview was constructed using the reference map deposited in the KEGG database (Fig. 5). Eighteen differential metabolites of 14 differentially enriched metabolic pathways present in the *B*. *dorsalis* larvae are worthy of attention. We summarized these differentially enriched metabolic pathways into amino acids, carbohydrates, nucleosides, and vitamin and cofactor metabolism.

**Figure 6:**
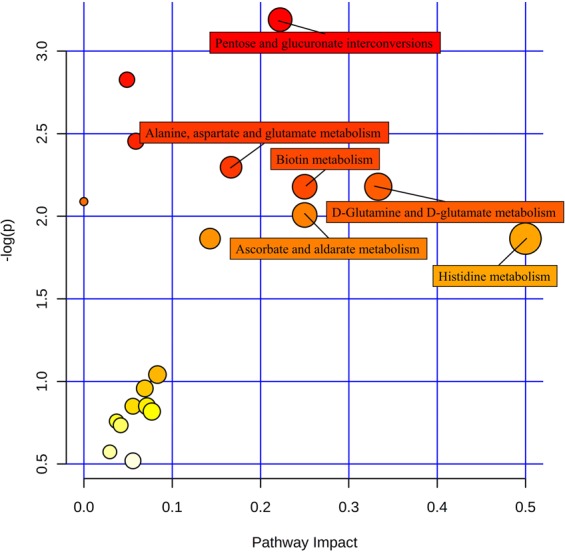
Metabolome map of significant metabolic pathways characterized in *B*. *dorsalis* larvae for the CK and Tr groups. Significantly changed pathways based on enrichment and topology analysis are shown. The x-axis represents pathway enrichment, whereas the y-axis represents pathway impact. Large sizes and dark colors represent major pathway enrichment and high pathway impact values, respectively.

Among these differentially enriched metabolic pathways, seven had pathway impact values exceeding 0.1, which was the threshold for relevance after the pathway enrichment and topology analysis. On the basis of the negative log(*P*) and impact values, we characterized histidine metabolism, d-glutamine and d-glutamate metabolism, biotin metabolism, ascorbate and aldarate metabolism, pentose and glucuronate interconversions, and alanine, aspartate, and glutamate metabolism in *B*. *dorsalis* larvae as the significantly relevant pathways affected by azadirachtin; their impact values were 0.5, 0.33, 0.25, 0.25, 0.22, and 0.17, respectively (Fig. 6).

## Discussion

In this study, azadirachtin mixed in an artificial diet was found to significantly prolong the developmental duration of larvae and decrease the larval survival rate and pupal weight. Although this situation is quite different from practical application in the field because *B*. *dorsalis* larvae are inside the fruit, and there is lack of evidence that azadirachtin penetrate the skin and flesh, the metabolomics analysis provides new insights into the biochemical response of *B*. *dorsalis* larvae to azadirachtin.

Azadirachtin could significantly reduce the quantity and relative composition of fatty acids^20^. However, only six lipids or lipid-like molecules were found to be differentially abundant in this study, and none of the lipid metabolism pathways were enriched. Hence, the targeted metabolomics for fatty acids or lipidomics should be considered to explore a more comprehensive effect of azadirachtin on the lipids of *B*. *dorsalis* larvae.

Azadirachtin was found to affect the carbohydrates metabolism of *B*. *dorsalis* larvae. Succinic and malic acids were the two differentially enriched metabolites in TCA cycles in this study. The TCA cycle, known as the citric acid cycle, has two important functions. The first involves the intermediate compounds for the synthesis of amino and fatty acids. The other involves the formation of large quantities of ATP, which provides energy for various synthetic processes^29^. The downregulation of such metabolites in *B*. *dorsalis* larvae is expected to indicate that a shortage of intermediate compounds and energy in the *B*. *dorsalis* larvae fed with azadirachtin diet. Xylitol can be used in the TCA cycle and after conversion into xylulose and arabitol through pentose and glucuronate interconversions (https://www.genome.jp/dbget-bin/www_bget?pathway:dme00040). So changes in the relative content of these sugars is expected to impact the generation of energy and intermediate compounds to maintain normal biological processes.

Azadirachtin was also found to affect the amino acid metabolism of *B*. *dorsalis* larvae. Glutamic acid is involved in many biochemical pathways and is regarded as a key metabolite linking carbon and nitrogen metabolism^30^. The relative high content of glutamic acid in treated larvae could be caused by histidine metabolism, d-glutamine and d-glutamate metabolism, and alanine, aspartate, and glutamate metabolism, through which nitrogenous metabolites are further converted into TCA cycles to make up the shortage of energy and intermediate compounds. As histidine regularly plays a key role in the active sites of enzymes^31^, it has to be maintained at a relative high content to meet the needs of enzyme reactions in *B*. *dorsalis* larvae fed with azadirachtin diet.

Azadirachtin also affected the vitamins and cofactor metabolism of *B*. *dorsalis* larvae, including biotin, also known as vitamin B7 or vitamin H. The relatively high content of biotin in treated larvae in this study could be related to the critical roles this cofactor plays in the intermediate metabolism of gluconeogenesis, fatty acid synthesis, and amino acid catabolism^32^. Myo-inositol is regarded as a vitamin-like essential nutrient^33^. Due to the shortage of intermediate compounds and energy caused by downregulation of succinic and malic acids, myo-inositol was converted into TCA cycles through galactose metabolism, thereby causing its relative low content in this study. Myo-inositol could also be partially responsible for poor growth of *B*. *dorsalis* larvae fed with azadirachtin diet, since its deficiency could result in inefficiency in digestion and food utilization and poor growth in shrimps and fish^34^.

## Conclusions

Although it is inappropriate to extrapolate the bioactivity results in this study to the project of *B*. *dorsalis* pest management, the integrated metabolomics analyses have revealed that azadirachtin has a significant effect on the carbohydrates, amino acids, and vitamin and cofactors metabolism of *B*. *dorsalis* larvae. This provides new insights into the mode of azadirachtin, the main active ingredient in neem based pesticides.

## Materials and Methods

### Chemicals and reagents

Methanol of HPLC grade was obtained from Tianjin Kermel Chemical Reagent Co., Ltd. (Tianjin, China). Chloroform and acetone were of analytical grade and obtained from the National Pharmaceutical Group (Shanghai, China). The ultrapure water was prepared by ELGA LabWater system. Pyridine, methoxylamine hydrochloride, and N,O-bis(trimethylsilyl)trifluoroacetamide (BSTFA, 98%) with trimethylchlorosilane (TMCS, 1%) were obtained from Aladdin (Shanghai, China). Azadirachtin (>90%) was kindly provided by associate Professor Yong-Qing Tian of the Key Laboratory of Natural Pesticide and Chemical Biology of the Ministry of Education of South China Agricultural University.

### Experimental procedures and bioactivities

The population of *B*. *dorsalis* was maintained in a laboratory under 25 ± 1 °C, 16:8 h light:dark cycle, and 70–80% RH. The artificial larval diet consisted of corn flour, yeast, sucrose, paper towel, hydrochloric acid, sodium benzoate, banana, and water, whereas the adult diet consisted of water, yeast, and sugar^35,36^.

These experiments contained two groups, namely, control (CK) and 1 μg/g azadirachtin treatment (Tr). The effects of azadirachtin on larval survival, developmental duration, and pupal weight were determined in accordance with a previous report and were briefly summarized below^37^. The stock solution of 10,000 μg/mL azadirachtin was prepared with acetone and stored at 4 °C. For the Tr group, the accurate volume of stock solution was added and fully mixed to ensure that the final azadirachtin concentration in the artificial larval diet was 1 μg/g. The same volume of acetone was added to the CK group. *B*. *dorsalis* eggs collected on the same day were counted under a microscope. Three hundred eggs of each replicate were transferred to the diet to investigate larval survival, developmental duration, and pupal weight. Treatments for each group were performed in triplicate.

### Sample collection

At the last instar of *B*. *dorsalis* larvae (8 days after inoculation), more than 100 larvae were collected and divided into two samples from each replicate of each group. These twelve samples were then snap-frozen in liquid nitrogen and stored at −80 °C. The stored larvae samples were ground into fine powder in liquid nitrogen and freeze-dried for 24 h until extraction.

### Metabolite extraction, derivatization, and analysis for GC–MS

The metabolite extraction method was in reference to a published procedure and described briefly as below^38^. Approximately 50 mg of lyophilized larvae powder from each sample was homogenized for 2 min with 300 µL of precooled solvent (V_(chloroform)_:V_(methanol)_:V_(water)_ = 1:2.5:1) for metabolite profiling analysis on GC–MS. The samples were then centrifuged at 12,000 *g* for 15 min at 4 °C. A total of 250 µL of supernatant was transferred to a new centrifuge tube. The deposit was re-homogenized for 1 min after adding 300 µL of precooled methanol. Subsequently, the samples were centrifuged at 12,000 *g* for 15 min at 4 °C, and 250 µL of supernatant was incorporated into the first centrifuge tube. The 500 µL combined supernatant was centrifuged at 12,000 *g* for 15 min at 4 °C. Finally, a total of 450 µL of supernatant was transferred to another new centrifuge tube to make QC and experimental samples.

All samples, including the QC samples, were nitrogen-dried in GC vials at room temperature. The residue was derivatized using a two-step procedure. First, 80 µL of methoxyamine (20 mg/mL in pyridine) was added to the vial and kept at 30 °C for 90 min. Second, 80 µL of BSTFA (1% TMCS) was added to the vial and maintained at 70 °C for 60 min^39^.

The 1 μL of derivatized sample was subjected to Agilent 7890 A/5975 C GC–MS system. It was analyzed in splitless mode with an HP-5MS capillary column (5% phenylmethylsiloxane: 30 m × 250 μm internal diameter, 0.25 μm thickness; Agilent, J & W Scientific, Folsom, CA, USA). The parameters for GC–MS analysis were set in reference to a previous study^40^. First, helium as the carrier gas at a constant flow rate of 1.0 mL/min. Second, the instrument was kept at 50 °C for 1 min, ramped to 100 °C at a rate of 10 °C/min for 1 min, ramped to 200 °C at a rate of 10 °C/min for 1 min, ramped to 280 °C at a rate of 10 °C/min for 1 min, and ramped to 320 °C at a rate of 10 °C/min for 1 min. Third, the injector, transfer line, and ion source were set at 250 °C, 280 °C, and 230 °C, respectively. Finally, mass spectra were acquired with electron ionization mode (70 eV) in full scan mode (m/z 50–650) and solvent delay was set at 5 min. The QC samples were acquired to evaluate stability during analysis. A saturated n-alkane mixture from C7 to C34 was also run at the beginning of the experimental work to determine retention indexes (RIs).

### Metabolite extraction and analysis for LC–MS

Approximately 25 mg of lyophilized larvae powder from each sample was homogenized for 5 min with 800 μL of precooled solvent (V_(methanol)_:V_(water)_ = 1:1) for metabolite profiling analysis by LC–MS. The samples were then centrifuged at 30,000 *g* for 20 min at 4 °C. Approximately 650 μL of supernatant was transferred to a new 1.5 mL polypropylene tube and then centrifuged at 25,000 *g* for 20 min at 4 °C. Next, 550 μL of supernatant was transferred into another new 1.5 mL polypropylene tube to make a QC sample, which was prepared by pooling the same volume of supernatant from each of the samples.

The parameters for LC–MS analysis were set with reference to literature, with some modifications^41^. The samples were subjected to a 2777 C UPLC (Waters, UK) coupled with an Xevo G2 XS QTOF high-resolution tandem mass spectrometer (Waters, UK). The injection volume was 10 μL. Seperation was performed on a HSS T3 C18 column (100 mm × 2.1 mm × 1.8 μm, Waters). The column oven was maintained at 50 °C. The mobile phase consisted of solvents A (water + 0.1% formic acid) and B (acetonitrile + 0.1% formic acid), with a flow rate of 0.4 mL/min. The gradient elution program was set as follows: 0–2 min, 100% A; 2–11 min, 0% to 100% B; 11–13 min, 100% B; and 13–15 min, 0% to 100% A. The Q-TOF mass spectrometer was operated in both positive and negative ion modes. For positive ion mode (ESI+), the detection parameters were set as capillary voltage: 3.0 kV; sampling cone voltage: 40 V; ESI source temperature: 120 °C; desolvation temperature: 450 °C; desolvation gas: 800 L/h; cone gas: 50 L/h; source offset: 80; TOF acquisition mode: sensitivity; acquisition method: continuum MS^E^ mode; TOF mass range: 50–1200 Da; scan time: 0.2 s; and collision energy function 2: trap CE ramp 20–40 eV. For negative ion mode (ESI−), the capillary voltage and desolvation temperature were set at 2.0 kV and 350 °C, respectively. The other parameters were set the same as those in the positive ion mode. During the acquisition, the leucine enkephalin signal was acquired every 3 s to calibrate the mass accuracy. Furthermore, QC samples were acquired to evaluate the stability of the LC–MS analysis during the whole acquisition.

### Data pre-processing and multivariate pattern recognition

The original GC–MS data were automatically analyzed using the automatic mass spectral deconvolution and identification system (AMDIS) and identified by comparing to the database of Fiehn, Golm, and NIST14^42,43^. The RI and mass similarity match were considered in metabolite identification. If the total match factor was greater than 80, then the metabolite identification was reliable^44,45^. Subsequently, the AMDIS output files were extracted and processed using MET-IDEA to obtain the final data set, which included sample information, metabolites, and their intensities^46^. Peaks caused by column bleeding and the BSTFA derivatization procedure were removed.

The original LC–MS data were converted into.abf files by using ABF converter software (https://www.reifycs.com/AbfConverter/). MS DIAL software (http://prime.psc.riken.jp/Metabolomics_Software/MS-DIAL/index.html) with the LC–MS/MS spectral database from MassBank of North America (http://mona.fiehnlab.ucdavis.edu/) was used for peak exaction, data baseline filtering, baseline calibration, peak alignment, deconvolution analysis, peak identification, and peak integration^47,48^. The metabolites were filtered by removing exogenous compounds, such as drugs or compounds of plant origin, on the basis of their likelihood to be present in biological samples^49^.

Total area normalization was performed to reduce the systematic biases within the experiment^50,51^. Data were log transformed and Pareto scaled for multivariate analysis to remove the offsets and adjust the importance of high and low abundance metabolites to an equal level^52^. Multivariate statistical analysis was performed using SIMCA 14.1 demo (Umea, Sweden). PCA showed the distribution of the original data. Supervised PLS-DA was applied to obtain a high level of group separation and to identify the variables responsible for classification^53^. The PLS-DA model was validated using sevenfold cross validation. The model quality was assessed based on R^2^ and Q^2^ scores, and the permutation test was conducted to further validate this model^34^. The PLS-DA model was used with the first principal component of VIP values combined with Student’s *t*-test to determine significantly differentially abundant metabolites between CK and Tr. The *q* values (adjusted *P* values), which were raw *P* values from the *t*-test adjusted using the Benjamini and Hochberg procedure (BH method), were applied to correct for multiple comparisons^45^. The fold change in each metabolite abundance was calculated by comparing the mean values of the peak areas obtained from Tr and CK.

### Pathway analysis

Analysis of metabolic pathways affected by azadirachtin was performed using MetaboAnalyst 4.0^28^. This system is a free web-based tool that uses the high-quality KEGG metabolic pathway database as the backend knowledgebase. The hypergeometric test was used for over representation analysis and the out-degree centrality was used for pathway topology analysis. The significantly affected pathways were selected either by *P* values from pathway enrichment analysis or by impact values from pathway topology analysis^45,54^. The impact values exceeding 0.1 and the negative log(*P*) values exceeding 2.0 were set as the thresholds in this study.

## References

[1] Clarke AR (2005). Invasive phytophagous pests arising through a recent tropical evolutionary radiation: The *Bactrocera dorsalis* complex of fruit flies. Annu. Rev. Entomol..

[2] Mulla MS, Su T (1999). Activity and biological effects of neem products against arthropods of medical and veterinary importance. J. Am. Mosquito Contr..

[3] Schmutterer H (1990). Properties and potential of natural pesticides from the neem tree, *Azadirachta indica*. Annu. Rev. Entomol..

[4] Martínez-Romero A, Ortega-Sánchez JL, Hernández-González SI, Olivas- Calderón EH, Alba-Romero JJ (2016). Application of neem tree in agriculture, industry, medicine, and environment: A review. Afr. J. Tradit. Complement. Altern. Med..

[5] Silva MA, Bezerra-Silva GCD, Vendramim JD, Mastrangelo T, Forim MR (2013). Neem derivatives are not effective as toxic bait for Tephritid fruit flies. J. Econ. Entomol..

[6] Chen C, Dong Y, Cheng L, Hou RF (1996). Deterrent effect of neem seed kernel extract on oviposition of the oriental fruit fly (Diptera: Tephritidae) in Guava. J. Econ. Entomol..

[7] Singh S, Singh RP (1998). Neem (*Azadirachta indica*) seed kernel extracts and azadirachtin as oviposition deterrents against the melon fly (Bactrocera curcurbitae) and the Oriental fruit fly (*Bactrocera dorsalis*). Phytoparasitica.

[8] Khan M, Aftab Hossain M, Saidul Islam M (2007). Effects of neem leaf dust and a commercial formulation of a neem compound on the longevity, fecundity and ovarian development of the melon fly, Bactrocera cucurbitae (Coquillett) and the oriental fruit fly, *Bactrocera dorsalis* (Hendel) (Diptera: Tephritidae). Pak. J. Biol. Sci..

[9] Singh S (2003). Effects of aqueous extract of neem seed kernel and azadirachtin on the fecundity, fertility and post-embryonic development of the melonfly, Bactrocera cucurbitae and the oriental fruit fly, *Bactrocera dorsalis* (Diptera: Tephritidae). J. Appl. Entomol..

[10] Schmutterer, H. The neem tree, *Azadirachta indica* a. Juss. And other meliaceous plants., MumbaiMumbai: Neem Foundation, 1995.

[11] Sengottayan, S. Physiological and biochemical effect of neem and other Meliaceae plants secondary metabolites against Lepidopteran insects. *Front Physiol* **4** (2013). 10.3389/fphys.2013.00359 PMC3868951 24391591

[12] Luntz AJM, Nisbet AJ (2000). Azadirachtin from the neem tree *Azadirachta indica*: Its action against insects. Anais da Sociedade Entomológica do Brasil.

[13] Lai D, Jin X, Wang H, Yuan M, Xu H (2014). Gene expression profile change and growth inhibition in Drosophila larvae treated with azadirachtin. J. Biotechnol..

[14] Wang H, Lai D, Yuan M, Xu H (2014). Growth inhibition and differences in protein profiles in azadirachtin‐treated *Drosophila melanogaster* larvae. Electrophoresis.

[15] Banerjee S, Rembold H (1992). Azadirachtin a interferes with control of serotonin pools in the neuroendocrine system of locusts. Naturwissenschaften.

[16] Kumar TR, Emerald DM, Prabu SM (2008). Effect of phytopesticide neem gold on protein metabolism in the fat body, testes, seminal vesicle and MARGs of adult male *Odontopus varicornis* (Dist.) (Hemiptera: Pyrrhocoridae). Biochemical and Cellular Archives.

[17] Babu R, Murugan K, Kavitha R (1997). Impact of azadirachtin on quantitative protein and lipid profiles during gonadotropic period of *Atractomorpha crenulata* Fab. (Orthoptera: Acrididae). Indian J. Exp. Biol..

[18] Rharrabe K, Amri H, Bouayad N, Sayah F (2008). Effects of azadirachtin on post-embryonic development, energy reserves and α-amylase activity of *Plodia interpunctella* Hübner (Lepidoptera: Pyralidae). J. Stored Prod. Res..

[19] Amirmohammadi F, Sendi JJ, Zibaee A (2012). Biomonitoring of the genotoxic and oxidative the effect of neemon mortality and physiological indices of *Hyphantria cunea* (Drury) (Lepidoptera). Munis Entomology & Zoology.

[20] Huang Z, Zhao M, Shi P (2012). Sublethal effects of azadirachtin on lipid metabolism and sex pheromone biosynthesis of the Asian corn borer *Ostrinia furnacalis*. Phytoparasitica.

[21] Khosravi R, Sendi JJ (2013). Effect of neem pesticide (Achook) on midgut enzymatic activities and selected biochemical compounds in the hemolymph of lesser mulberry pyralid, *glyphodes pyloalis* walker (Lepidoptera: Pyralidae). J. Plant Prot. Res..

[22] Klassen, A. *et al*. Metabolomics: Definitions and significance in systems biology, in: *Metabolomics: From Fundamentals to Clinical Applications* (ed. Sussulini A.) 3–17 (Springer International Publishing, 2017). 10.1007/978-3-319-47656-8_1 28132174

[23] Aliferis KA, Jabaji S (2011). Metabolomics - a robust bioanalytical approach for the discovery of the modes-of-action of pesticides: A review. Pestic. Biochem. Phys..

[24] Lu Y, Qi Z, Xu W (2014). Global metabolomic analyses of the hemolymph and brain during the initiation, maintenance, and termination of pupal diapause in the cotton bollworm, *Helicoverpa armigera*. PLoS One.

[25] Qu LJ (2014). Radiation-induced metabolomic changes in sterile male *Monochamus alternatus* (Coleoptera: Cerambycidae). J. Insect Sci..

[26] Killiny N (2017). Metabolomic analyses of the haemolymph of the Asian citrus psyllid *Diaphorina citri*, the vector of huanglongbing. Physiol. Entomol..

[27] Eriksson, L., Byrne, T., Johansson, E., Trygg, J. & Vikström, C. Multi-and megavariate data analysis basic principles and applications, Umetrics Academy, 2013).

[28] Chong J (2018). MetaboAnalyst 4.0: Towards more transparent and integrative metabolomics analysis. Nucleic Acids Res..

[29] Pallardy, S. G. CHAPTER 6 - Enzymes, Energetics, and Respiration, in: *Physiology of Woody Plants* (*Third Edition*) (ed. Pallardy S. G.) 169–197 (Academic Press, 2008).

[30] Shen, J. Chapter 2.4 - Glutamate, in: *Magnetic Resonance Spectroscopy* (ed. Stagg C. & Rothman D.) 111–121 (Academic Press, 2014).

[31] Lea, P. J. & Azevedo, R. A. PRIMARY PRODUCTS | amino acids, in: *Encyclopedia of Applied Plant Sciences* (ed. Thomas B.) 871–883 (Elsevier, 2003).

[32] Dasgupta, A. Chapter 2 - Biotin: Pharmacology, Pathophysiology, and Assessment of *Biotin Status*, *in: Biotin and Other Interferences in Immunoassays* (ed. Dasgupta A.) 17–35 (Elsevier, 2019).

[33] Combs, G. F. & McClung, J. P., Chapter 19 - Vitamin-Like Factors, in: *The Vitamins (Fifth Edition)* (ed. Combs G. F. & McClung J. P.) 453–498 (Academic Press, 2017).

[34] Yang C (2018). GC–TOF/MS-based metabolomics studies on the effect of protein sources in formulated diet for pearl oyster *Pinctada fucata martensii*. Aquaculture.

[35] Cheng D (2017). Gut symbiont enhances insecticide resistance in a significant pest, the oriental fruit fly *Bactrocera dorsalis* (Hendel). Microbiome.

[36] Zhao X (2018). The divergence in bacterial components associated with *Bactrocera dorsalis* across developmental stages. Front Microbiol.

[37] Khaeso K (2018). Assessing the effects of gut bacteria manipulation on the development of the oriental fruit fly, *Bactrocera dorsalis* (Diptera; Tephritidae). Symbiosis.

[38] Zhou Y (2020). The comparative metabolic response of *Bactrocera dorsalis* larvae to azadirachtin, pyriproxyfen and tebufenozide. Ecotox. Environ. Safe..

[39] Wu Y (2012). The metabolic responses to aerial diffusion of essential oils. PLoS One.

[40] Zhao LJ (2016). Changes in metabolites in maize seedlings under chlorsulfuron and cadmium stress. J. Agr. Sci..

[41] Wu J (2017). Brain metabolomic profiling of eastern honey bee (*Apis cerana*) infested with the mite *Varroa destructor*. PLoS One.

[42] Kind T (2009). FiehnLib: Mass spectral and retention index libraries for metabolomics based on quadrupole and Time-of-Flight gas Chromatography/Mass spectrometry. Anal. Chem..

[43] Hummel, J., Selbig, J., Walther, D. & Kopka, J. The Golm Metabolome Database: A database for GC-MS based metabolite profiling, in: *Metabolomics: A Powerful Tool in Systems Biology* (ed. Nielsen J. & Jewett M. C.) 75–95 (Springer Berlin Heidelberg, 2007).

[44] Luo Q, Sun L, Hu X, Zhou R (2015). The Variation of Root Exudates from the Hyperaccumulator *Sedum alfredii* under Cadmium Stress: Metabonomics Analysis. PLoS One.

[45] Wang L (2015). Reconstruction and analysis of correlation networks based on GC–MS metabolomics data for young hypertensive men. Anal. Chim. Acta.

[46] Lei Z, Li H, Chang J, Zhao PX, Sumner LW (2012). MET-IDEA version 2.06; Improved efficiency and additional functions for mass spectrometry-based metabolomics data processing. Metabolomics.

[47] Tsugawa H (2015). MS-DIAL: Data-independent MS/MS deconvolution for comprehensive metabolome analysis. Nat. Methods.

[48] Kind T (2018). Identification of small molecules using accurate mass MS/MS search. Mass Spectrom. Rev..

[49] Bernardo-Bermejo, S. *et al*. An untargeted metabolomic strategy based on liquid chromatography-mass spectrometry to study high glucose-induced changes in HK-2 cells. *J*. *Chromatogr*. *A* (2019). 10.1016/j.chroma.2019.03.009 30878178

[50] De Livera AM (2012). Normalizing and integrating metabolomics data. Anal. Chem..

[51] Want E, Masson P (2011). Processing and analysis of GC/LC-MS-based metabolomics data. Methods Mol. Biol..

[52] Ch R, Singh AK, Pandey P, Saxena PN, Reddy Mudiam MK (2015). Identifying the metabolic perturbations in earthworm induced by cypermethrin using gas chromatography-mass spectrometry based metabolomics. Sci. Rep..

[53] Yang C (2019). Response to different dietary carbohydrate and protein levels of pearl oysters (*Pinctada fucata martensii*) as revealed by GC–TOF/MS-based metabolomics. Sci. Total Environ..

[54] Wang X, Yang B, Sun H, Zhang A (2012). Pattern recognition approaches and computational systems tools for ultra performance liquid chromatography–mass Spectrometry-Based comprehensive metabolomic profiling and pathways analysis of biological data sets. Anal. Chem..

